# High risk HPV types 18 and 16 are potent modulators of oral squamous cell carcinoma phenotypes in vitro

**DOI:** 10.1186/1750-9378-2-21

**Published:** 2007-11-14

**Authors:** Nicole Reddout, Todd Christensen, Anthony Bunnell, Dayne Jensen, Devin Johnson, Susan O'Malley, Karl Kingsley

**Affiliations:** 1 Department of Biomedical Sciences, School of Dental Medicine, University of Nevada, Las Vegas, USA

## Abstract

**Background:**

Human papillomavirus (HPV) has been confirmed as the primary etiological factor that transforms cervical epithelia into cancer. The presence of HPV in oral cancers suggests that HPV may play a similar role in transforming the oral epithelia. A high degree of variability in the prevalence of HPV in oral cancers has been found, however, raising questions regarding its role in the transformation and development of oral cancers. The goal of this study was to test our hypothesis that high-risk HPV strains HPV16 and HPV18 will alter the phenotype of transformed oral squamous cell carcinoma cell lines, CAL27, SCC-15 and SCC-25 *in vitro*.

**Results:**

CAL27 cells transfected with HPV18, HPV16, as well as HPV16/18 co-transfectants, demonstrated significant increases in proliferation, adhesion and cell spreading compared with non-transfected controls. These observed differences were correlated with a small level of increased cell survival. SCC-15 cells, however, displayed a differential response to HPV transfection, with only HPV18-transfectants demonstrated changes to proliferation. Interestingly, SCC-25 cells displayed a more complex response, with HPV16-induced increases in cell proliferation, viability and cell spreading, while HPV18- and 16/18-transfectants exhibited reduced adhesion and proliferation.

**Conclusion:**

Determining the potential of specific high-risk HPV strains to alter phenotypic behaviors of already transformed oral carcinomas is a critical step in providing more accurate prognosis and treatment options for oral cancer patients. The identification of differential responses to specific HPV strains among oral cancers suggests a more significant, complex and multifactorial role of HPV, not only in transforming, but also in modulating, the phenotype and treatment responsiveness of precancerous and cancerous oral lesions. This study provides some of the first evidence to help identify the important molecular markers for pathways that could be used to determine the most effective and appropriate treatment plans for oral cancer patients with concomitant oral HPV infections.

## Background

Human papillomavirus (HPV) has been confirmed as the primary etiological factor that transforms cervical epithelia into cancer [1]. Certain HPV types are detected in virtually all invasive cervical cancer biopsies and have thus been designated as oncogenic or high-risk HPV [2,3]. According to epidemiological case-control studies [4], 15 high-risk HPV types have been acknowledged (types 16, 18, 31, 33, 35, 39, 45, 51, 52, 56, 58, 59, 68, 73, and 82), while 3 types have been designated as probable high-risk (types 26, 53, and 66) and 12 types have been classified as low-risk.

Of these high-risk HPV types, HPV16 is the most common strain present in biopsies from women with cervical squamous cell carcinoma (SCC) and it is also the most common strain of high-risk HPV from biopsies of head and neck SCC (HNSCC) [3,5,6]. HPV18 is the second most commonly found high-risk HPV strain in both invasive cervical cancer [7] and HNSCC [3]. Other common high-risk cervical cancer HPV strains are rarely, or never, detected in oral SCC biopsies [3,8].

Infection of cervical SCC and HNSCC with high-risk HPV has thus been well documented. Co-infection of these cancers with more than one high-risk HPV type has also been documented, but it is not found as frequently as infections with single high-risk HPV strains [3,7]. When co-infected HNSCC specimens are identified, however, they are most often co-infected with HPV16 [3]. Co-infection of HNSCC with HPV16 and HPV18 has been documented in a relatively small proportion of samples from numerous studies [3,9-12].

Recent studies have provided further evidence that HPV is an independent risk factor for oral SCC, determining that HPV is found in three times as many precancerous oral mucosa, and almost five times as many oral cancers, compared with normal oral mucosa [3,5,12-16]. Despite the increased risk associated with these HPV infections, the comparatively low incidence of HPV16 and HPV18 in oral SCC, combined with an even lower prevalence of HPV in premalignant lesions, suggests that while these high risk HPV strains may induce transformation in some subset of oral cancers, they may infect and subsequently act to modulate phenotypes in established oral SCCs (OSCC) [8].

Much of the literature related to HPV infection and oral cancers involves retrospective analyses of tumor biopsies and epidemiologic studies. While these and other studies have informed our understanding of the roles of HPV in oral cancer, they have not adequately addressed the apparent contradictory evidence that HPV infection may not be causally related to the formation of all, or even most, oral carcinomas. This seemingly contradictory evidence may be explained by the inability of varied methods, such as PCR and other detection techniques, to distinguish between HPV infections causally related to cancer development and those that are concomitant, non-causal HPV infections [17,18]. By examining the effects of HPV on transformed OSCC *in vitro*, we may begin to elucidate and understand the etiologic factors that are necessary and sufficient to transform the oral mucosa and the factors that may promote proliferative potential in already transformed OSCC.

The pathway of HPV-induced transformation is well established in cervical SCC, with transformation attributable to the HPV Early (E) genes which code for proteins that, in addition to promoting viral replication, are capable of binding and inactivating transcription factors with tumor suppressor function, such as *p53 *and *Rb*, regulators of cell-cycle checkpoints at the G_1 _phase [19-21]. The actions of high-risk HPV gene products play a similar role in the carcinogenesis and tumor progression in oral cancer. [22].

Although some studies have demonstrated the transformation of human foreskin and cervical keratinocytes *in vitro *using HPV16 [23,24], until recently, the role of HPV in already transformed OSCC had not been investigated. We recently determined that the OSCC cell line, CAL27, transfected with HPV16, exhibited significantly increased proliferation, when compared with non-transfected controls [25]. This increased proliferation was observed even in the absence of serum, and the effects were specific to proliferation, adhesion, and morphology, but not to cell viability.

Based upon our previous studies and the mounting evidence of the possible role of HPV in modulating oral cancer phenotypes, we sought to determine if the observed effects of HPV16 on CAL27 can be generalized to other high-risk HPV strains and oral cancer cell lines. To this end, we examined the effects of the two strains of high-risk HPV most commonly associated with oral cancer, HPV16 and HPV18, both alone and in combination, on the proliferative phenotype of multiple OSCC cell lines *in vitro*. Based upon our previous findings that HPV16 induced increased proliferation [25], we hypothesized that infection with high risk HPV18 would result in similar phenotypic alterations in proliferation, viability, and morphology of three oral cancer cell lines, CAL27, SCC-15, and SCC-25. We further hypothesized that co-infection with HPV16 and HPV18 would also yield phenotypic changes similar to those observed for CAL27 infected with HPV16.

Our results provide one of the first demonstrations that the two high-risk HPV strains most commonly associated with oral SCC, HPV16 and HPV18, significantly affect the proliferative potential of multiple oral cancers *in vitro*. In particular, we have determined that among CAL27 cells, transfection with HPV16 and HPV18, and HPV16/18 co-transfection, exhibited measurable differences in adhesion, morphology, and proliferation, compared with non-transfected controls. SCC-15 cells, however, displayed differential responses to HPV; HPV18 and co-transfection increased cell proliferation, while no significant changes to viability or adhesion were observed. Moreover, SCC-25 cells also displayed a differential response to HPV strains. Specifically, HPV16 induced a significant increase in proliferation and cell spreading, while HPV18 and HPV16/18 significantly reduced adhesion, proliferation and cell spreading. Thus, the identification of differential responses to specific HPV strains among oral cancers may be the first step to identifying the important molecular markers and pathways that could be used to develop more effective and appropriate treatment plans for oral cancer patients with concomitant oral HPV infections.

## Results

### Proliferation

Our previous studies of OSCC proliferation of one OSCC cell line, CAL27, found that CAL27 cells proliferated more rapidly when plated at higher density, even in the absence of a serum stimulus. Furthermore, transfection of these cells with HPV16 further increased the proliferation rate of CAL27 cells at low density, with this specific effect further modulated in the presence of a serum stimulus [25]. Based upon these observations, we hypothesized that both of the most commonly found high risk oral HPV strains, HPV16 and HPV18, would similarly modulate the proliferation of oral cancers *in vitro*. To test our hypothesis, we expanded our study to include multiple OSCC cell lines, CAL27, SCC-15, and SCC-25, and also included both high risk oral HPV types, HPV16 and HPV18.

#### CAL27

**Figure 1:**
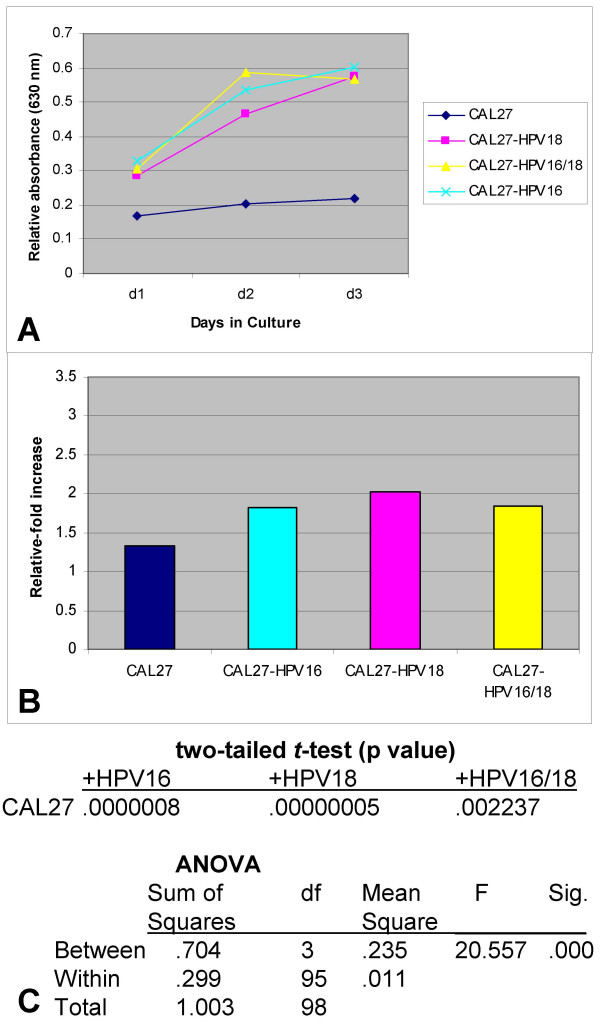
**Proliferation of CAL27 cells was increased by both HPV18 and HPV16 *in vitro***. HPV-transfected and control CAL27 cells were plated in 96-well assay plates with media containing 10% fetal bovine serum (FBS) and were allowed to proliferate for three days. (A) The addition of HPV16 and HPV18, either alone or in combination, was sufficient to stimulate the proliferation of CAL27 cells significantly (n = 96, p < 0.01). (B) Measurements of the relative change in proliferation between day 3 and day 1 revealed that the addition of HPV stimulated an increase in CAL27 proliferation from 1.3-fold to nearly 2.0-fold under each experimental condition. (C) Statistical tests between non-transfected and transfected cells revealed that the HPV-induced increases in proliferation were significant, verified using two-tailed *t*-tests and ANOVA.

CAL27 cells, CAL27 mock transfectants (mTF), and CAL27 transfectants with HPV16 (CAL27-HPV16), with HPV18 (CAL27-HPV18), and co-transfectants (CAL27-HPV16/18) were plated in media containing fetal bovine serum (FBS) and their proliferation was measured over three days in three separate, independent experiments. Our results demonstrated that the presence of HPV significantly increased proliferation of CAL27 cells over three days (n = 96, p < .01): HPV18 (+161%), HPV16 (+172%), HPV16 and HPV18 co-transfection (+160%) (Fig. 1A).

To reduce the proliferation-stimulating effects of trypsinizing and plating cells into each experimental assay, previously observed between day 0 and day 1 in most assays, we subsequently determined the relative change in proliferation between day 3 and day 1 (measured as day 3 minus day 1). Our results from this analysis revealed that the presence of HPV increased proliferation of CAL27 cells from a baseline relative-fold increase of 1.325 (CAL27, CAL27 mTF) to an approximate relative fold-increase of almost 2 for all HPV types tested (HPV18: 2.0-fold; HPV16: 1.8-fold; HPV16/18: 1.85-fold) (n = 96) (Fig. 1B).

In addition, we performed two-tailed *t*-tests to validate each HPV-induced increase in cellular proliferation in CAL27 cells. These calculations revealed that each experimental treatment represented a statistically significant increase in proliferation (HPV18, p < 0.01; HPV16, p < 0.01; HPV16/18, p < 0.01) but were not significantly different from one another (p > 0.05). Because these analyses involved multiple two-sample *t*-tests, the results have a higher probability of Type I error (incorrectly rejecting the null hypothesis, H_O_); therefore, ANOVA was performed to more accurately assess the relationship among groups. These results indicate a statistically significant difference between experimental groups, but not within groups (Fig. 1C), providing further validation of the statistical differences observed with the HPV-transfected CAL27 experimental cells.

#### SCC-15

**Figure 2:**
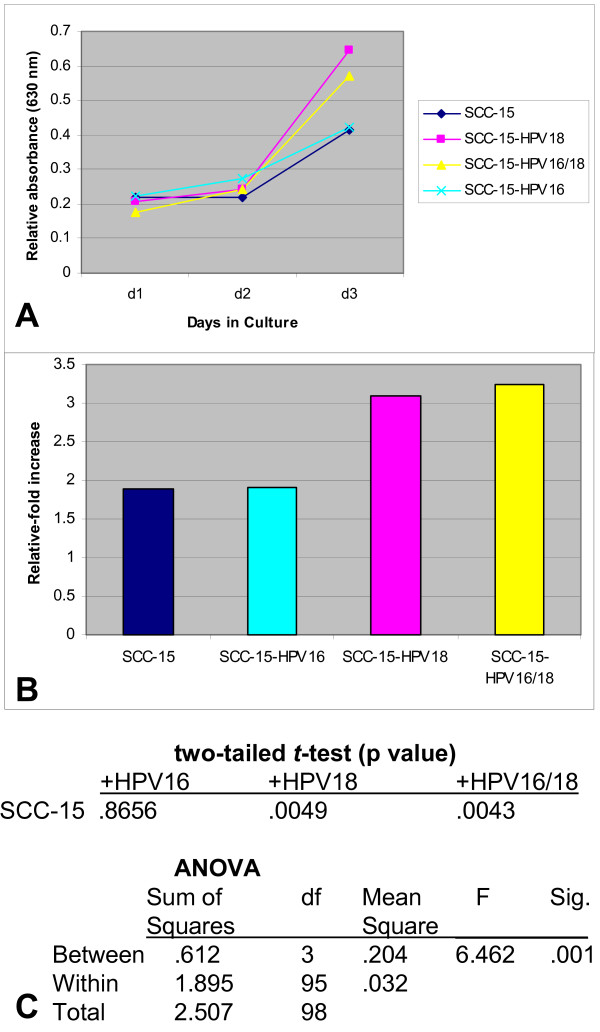
**Proliferation of SCC-15 cells was altered by HPV18, alone or in combination with HPV16, but not HPV16 alone, *in vitro***. HPV-transfected and control SCC-15 cells were plated in 96-well assay plates with media containing 10% FBS and were allowed to proliferate for three days. (A) HPV18- and HPV16/18 co-transfected SCC-15 cells exhibited increased proliferation (n = 120, p < 0.01) although HPV16-transfectants exhibited no deviation from control or mock-transfected cells. (B) Relative change in proliferation, measured between day 3 and day 1, revealed that proliferation of the control and HPV-16 transfectant was approximately 2-fold, while the proliferation of HPV18 and co-transfectants increased to approximately 3-fold. (C) Two-tailed *t*-tests and ANOVA were used to verify the statistical significance of HPV18 and co-transfectants and the lack of significance between controls and HPV16-transfected SCC-15 cells.

To further our understanding of oral cancer behavior, we expanded our analysis to include the OSCC line, SCC-15. SCC-15 cells, SCC-15 mock transfectants (mTF), and SCC-15 transfectants with HPV16 (SCC-15-HPV16), with HPV18 (SCC-15-HPV18), and co-transfectants (SCC-15-HPV16/18) were plated in media containing FBS and their proliferation was measured over three days in three separate, independent experiments. Our results demonstrated a differential behavioral response to the HPV strains tested (n = 96). Specifically, the presence of HPV18 and co-transfection with HPV18 and HPV16 significantly increased proliferation, increasing it by 55% and 37%, respectively, over non transfected controls (n = 72, p < 0.01). However, transfection with HPV16 did not produce a measurable effect on proliferation over the same time period (n = 48, p > 0.05) (Fig. 2A).

As described above, we determined the relative change in proliferation between day 3 and day 1 (day 3 minus day 1) for these cells. Our results from SCC-15 cells revealed that the presence of HPV18 increased proliferation of SCC-15 cells from a baseline relative-fold increase of 1.897 to a relative fold-increase of more than 3 with HPV18 (3.099-fold) and co-transfection, HPV18 and HPV16 (3.236), but not with exposure to HPV16 (1.900), which was virtually indistinguishable from non-transfected and mock transfected cells (n = 96) (Fig. 2B).

We subsequently performed two-tailed *t *tests to validate the differential HPV-specific responses observed in cellular proliferation of SCC-15 cells. These calculations revealed a statistically significant increase in proliferation with HPV18 (n = 48, p < 0.01) and HPV16/18 (n = 48, p < 0.01). However, SCC-15 response to HPV16 was not significantly different from non-transfected or mock transfected cells (n = 48, p > 0.05). ANOVA was performed to more accurately assess the relationship among groups. These results indicate a statistically significant difference between experimental groups, but not within groups (Fig. 2C).

#### SCC-25

**Figure 3:**
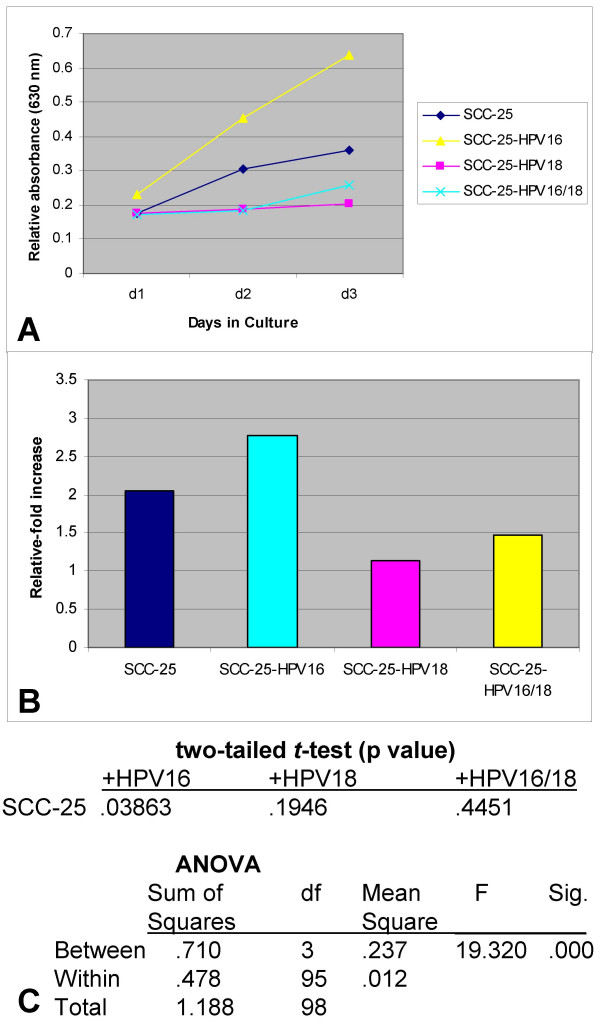
**Proliferation of SCC-25 cells was increased by HPV16 and decreased by HPV18 and co-transfection, *in vitro***. HPV-transfected and control SCC-25 cells were plated in 96-well assay plates with media containing 10% FBS and allowed to proliferate for three days. (A) HPV16-transfected cell proliferation was significantly enhanced, while HPV18- and HPV16/18-cell proliferation was slightly inhibited, compared with control cells (n = 96, p < 0.05). (B) The relative change in proliferation as measured between day 3 and day 1 revealed that baseline SCC-25 proliferation of 2.-fold was increased to approximately 3-fold by HPV16, while HPV18 and HPV16/18 reduced this rate to approximately 1- and 1.5-fold, respectively. (C) Two-tailed *t*-tests and ANOVA revealed that only HPV16-transfected SCC-25 cells were statistically different from controls.

We also evaluated the OSCC line, SCC-25, as outlined previously, and their proliferation was measured over three days in three separate, independent experiments. Our results once again demonstrated a differential behavioral response to the HPV strains tested (n = 96). For this cell line, however, only HPV16 significantly increased proliferation, 77% over non transfected controls (n = 72, p < 0.05). Interestingly, neither co-transfection with HPV16 and HPV18 nor HPV18 alone produced a measurable effect on proliferation over the same time period (n = 72, p > 0.05) (Fig. 3A).

We subsequently determined the relative change in proliferation between day 3 and day 1 and found that the presence of HPV16 increased proliferation of SCC-25 cells from a baseline relative-fold increase of 2.047 to a relative fold-increase of almost 3 (2.768-fold) with HPV16. Transfection with HPV18 resulted in a lower proliferation (1.142-fold increase, day3 minus day1) compared with controls, as did co-transfection with HPV16 and HPV18 (1.478-fold increase) (n = 96, p > .05) (Fig. 3B).

We subsequently performed two-tailed *t *tests to validate the differential HPV16-specific responses observed in cellular proliferation of SCC-25 cells. These calculations revealed a statistically significant increase in proliferation with HPV16 (n = 48, p < 0.05). However, SCC-25 response to HPV18 and HPV16 with HPV18, although comparatively less than non-transfected cells, was not significantly different from these controls (n = 96, p > 0.05). Furthermore, the results from the one-way ANOVA indicated a statistically significant difference between the HPV16 experimental group and all other groups (Fig. 3C).

### Viability

To evaluate if the HPV-specific changes in proliferation among these cell lines were related to changes in viability or cell survival, we determined the percentage of viable cells from each set of assays we performed (Table 1). Our results provide evidence that each cell line may display a characteristic response in viability that is HPV-strain specific. For example, CAL27 cell viability was measurably increased by HPV16 (+11%), HPV18 (+22%), and HPV16/18 (+8%). In addition, SCC-15 cell viability was only slightly altered by HPV16 (-2%) and HPV18 (-1%), but was drastically reduced by HPV16/18 co-transfection (-44%). Finally, SCC-25 cell viability also displayed a differential response to HPV16 (+6%), but not to HPV18 (-1%) or HPV16/18 (+2%).

**Table 1:** HPV effects on oral cancer viability *in vitro*. HPV strains induced differential responses among CAL27, SCC-15 and SCC-25 cell lines. CAL27 viability was increased under all HPV experimental conditions, while SCC-15 and SCC-25 exhibited more nuanced, differential responses in cell viability to the HPV strains tested.

**Cell viability**	**no HPV**	**HP16**	**HPV18**	**HPV16/18**
CAL27 (% change)	73%	84% **(+11%)**	95% **(+22%)**	81% **(+8%)**
SCC-15 (%change)	89%	87% (-2%)	88% (-1%)	45% **(-44%)**
SCC-25 (% change)	80%	86% **(+6%)**	79% (-1%)	82% (+2%)

### Adhesion

**Figure 4:**
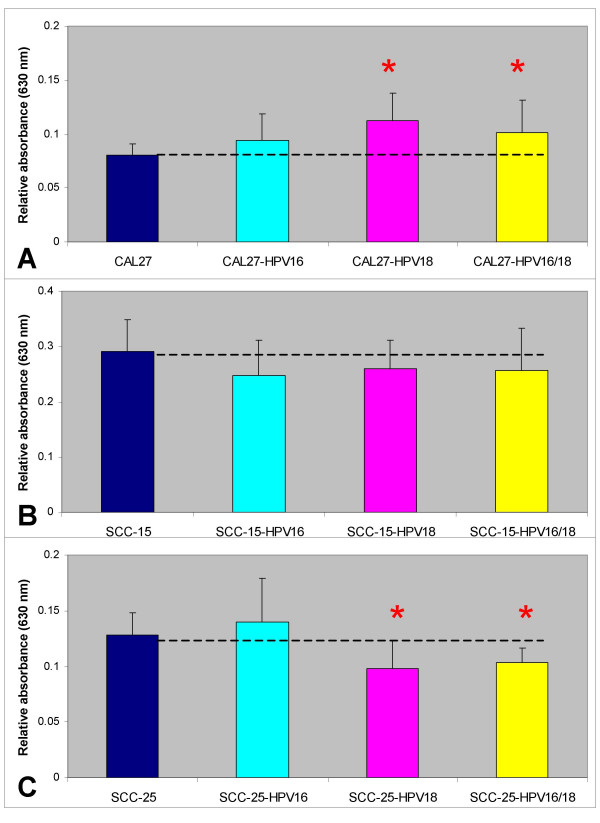
**Cell adhesion was altered by HPV18 and co-transfection of HPV16/18 in CAL27 and SCC-25 cells**. (A) CAL27 adhesion was increased by HPV16 (+18%), HPV18 (+40%) and HPV16/18 (+26%) although only the increases among HPV18 and HPV16/18-transfectants were significant (n = 32, p < 0.01), as measured by 30-minute *in vitro *adhesion assays. (B) SCC-15 adhesion was slightly reduced by HPV16 (-15%), HPV18 (-11%) and HPV16/18 (-12%), although these differences were not significant (n = 48, p > 0.05). (C) SCC-15 adhesion was increased slightly by HPV16 (+8%) but was decreased significantly by HPV18 (-24%) and HPV16/18 (-20%) (n = 32, p < 0.01).

Based upon the results from cell proliferation and viability assays, we sought to determine if these variable responses might also correlate with measurable alterations in cellular adherence as measured by 30-minute *in vitro *adhesion assays (Fig. 4). These analyses revealed that baseline CAL27 cellular adhesion was also measurably increased by all HPV treatments; HPV16 (+18%; n = 16, p = 0.05), HPV18 (+40%; n = 16, p < 0.01), and HPV16/18 (+26%, n = 16, p = 0.01), although the increase in CAL27 adhesion with HPV16 was not statistically significant (Fig. 4A).

SCC-15 adhesion, however, was not significantly impacted by the presence of HPV, although adhesion was slightly lower in each HPV-treatment category than in the non-transfected controls (Fig. 4B); HPV16 (-15%, n = 16, p = 0.06), HPV18 (-11%, n = 16, p = 0.12) and HPV16/18 (-12%, n = 16, p = 0.17). Interestingly, SCC-25 cell adhesion displayed a differential response to HPV treatment, increasing with HPV16, although not significantly (+8%, n = 16, p = .323), but decreasing significantly with HPV18 (-24%, n = 16, p < 0.01) and HPV16/18 (-20%, n = 16, p < 0.01) (Fig. 4C).

### Morphology and cell spreading

**Figure 5:**
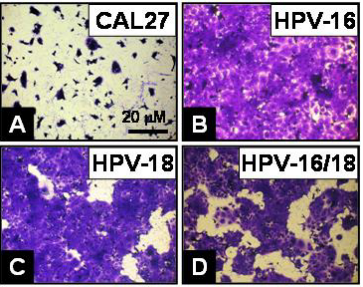
**CAL27 morphology was altered by the presence of HPV**. CAL27 cells (A) were plated in 96-well plates and allowed to proliferation with 10% FBS for three days. (B) HPV16-, (C) HPV18- and (D) HPV16/18-transfected cells increased in absolute number, confluence, and extent of cell spreading than non-transfected or mock-transfected cells (data not shown) under these conditions.

Guided by the results of proliferation, viability and adhesion data, we hypothesized that the most striking changes in cellular morphology and cell spreading would be evident between the CAL27 non-transfected and HPV-transfectants (Fig. 5). These images demonstrated the increase in both the absolute number and extent of spreading and confluence among the HPV16 (Fig. 5B), HPV18 (Fig. 5C), and HPV16/18 CAL27 (Fig. 5D), compared with CAL27 non-transfected controls (Fig. 5A).

**Figure 6:**
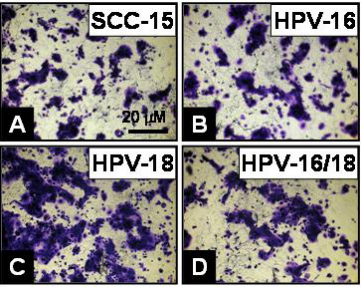
**SCC-15 morphology was not significantly altered by HPV**. SCC-15 cells (A) were plated in 96-well plates and allowed to proliferation with 10% FBS for three days. HPV-transfected cells (B-D) did not appear to exhibit dramatic and significant alterations in cell spreading or confluence by HPV treatments.

However, the effects of HPV on SCC-15 cells, although visible, were less prominent (Fig. 6). For example, although the absolute number of HPV18-transfected SCC-15 cells was comparatively greater (Fig. 6C), almost all cells from the HPV16 (Fig. 6B), HPV16/18 (Fig. 6D), and non-transfected control groups (Fig. 6A) exhibited nearly uniform spreading.

**Figure 7:**
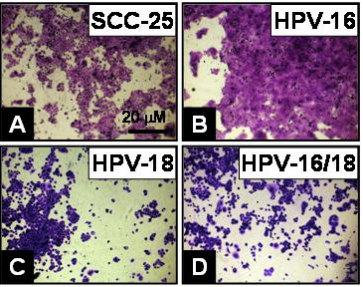
**SCC-25 morphology displayed differential responses to HPV**. SCC-25 cells (A) were plated in 96-well plates and allowed to proliferation with 10% FBS for three days. (A) HPV16-transfected cells exhibited a profound increase in absolute number, confluence and cell spreading, while (B) HPV18- and (C) HPV16/18-transfected cells demonstrated visible decreases in cell number, spreading and confluence by day 3.

SCC-25 cells, however, demonstrated a greater differential response to HPV than either CAL27 or SCC-15 cells (Fig. 7). SCC-25 cell proliferation (Fig. 7A) was conspicuously increased by the addition of HPV16 to this cell line (Fig. 7B). Moreover, as was also observed in the proliferation assays. HPV18 (Fig. 7C) and HPV16/18 co-transfectant (Fig. 7D) proliferation and spreading were noticeably inhibited in these groups.

### Transfection

**Figure 8:**
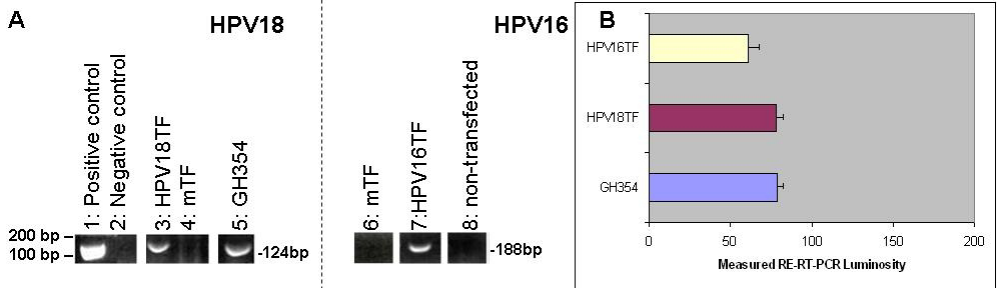
**HPV mRNA was expressed *in vitro *only after transfection**. (A) RT-PCR using total RNA collected from CAL27, SCC-15 and SCC-25 non-transfectant cells does not express HPV-mRNA (representative sample of CAL27, screened for HPV16: lane 8). RT-PCR confirmed the expression of HPV18-mRNA (lane 3) and HPV16-mRNA (lane 7) from all samples (CAL27 data shown). (B) Scanning densitometry measurement of relative endpoint RT-PCR band intensities from endogenous HPV (GH354: A, lane 5) were compared to HPV16, HPV18 and HPV16/18 co-transfectants, which revealed that HPV mRNA expression in all transfectants was roughly equivalent in all samples analyzed.

To determine if the differential effects of HPV on these cell lines was, in part, due to the efficiency of the transfection, we measured HPV16, HPV18, and HPV16/18 mRNA from each cell line prior to, and following, HPV transfection. RT-PCR from total RNA confirmed the expression of HPV18 and HPV16 (Fig. 8A) from randomly selected experiments. Densitometry measurements from multiple ethidium bromide (EtBr) band intensities, following relative endpoint RT-PCR, confirmed transfection efficiency and revealed comparable HPV expression levels between an endogenously-expressed HPV strain (GH354 cells) and HPV-transfected cells from these experiments (Fig. 8B).

## Discussion

Infection with high-risk HPV has been implicated in nearly all cases of cervical intraepithelial neoplasia and invasive squamous cell carcinomas [23,26,27], and the presence of HPV DNA in oral leukoplakias and oral malignant lesions suggests that HPV may also function to transform the oral mucosa. However, the comparatively low presence of HPV in pre-malignant oral lesions, combined with evidence that HPV is present only in a subset of oral cancers, implies that HPV infection may instead act to mediate oral cancer phenotypes in some cases, and possibly contributes to the malignant or transformation process rather than being a primary etiologic factor inducing oral carcinogenesis. We recently reported some of the first evidence that a high-risk HPV strain, HPV16, induced significant phenotypic changes in an already transformed OSCC line, CAL27 [25] reported significant increases in proliferation and changes to cell morphology.

Although HPV16 is the most common high-risk HPV strain in both cervical SCC and OSCC, another high-risk HPV strain, HPV18, it is also found in OSCC, either alone or in combination with HPV16. Based upon these observations, we hypothesized that, HPV16 and HPV18, alone or in combination, would be sufficient to induce significant, measurable alterations to oral cancer phenotypes, particularly to proliferation and cell morphology. Expanding our study parameters to encompass multiple OSCC cell lines, including not only CAL27, but also SCC-15 and SCC-25, we sought to test these hypotheses. Our results from these expanded studies, however, demonstrated instead that HPV induced differential responses between each of these OSCC cell lines, suggesting the presence of more complex and dynamic relationships among these variables.

That both of these high-risk, most commonly identified oral HPV strains, do not elicit equivalent responses in all cell lines tested is likely because oral cancers, similar to most other cancers, are not dependent upon a single receptor or signal transduction pathway for growth, development, or progression [28]. These results underscore the complicated nature of the mechanisms or pathways that may be common, or ubiquitous, to oral cancers and that may be potential therapeutic or prevention targets, without significant overlap with pathways necessary for maintenance of healthy cells and tissues.

These data combined suggest that understanding the critical differences that exist between oral and cervical carcinogenesis, may provide some insight into the mechanisms of phenotypic alteration that have been observed in this study. For example, it has been observed that while the *p53 *gene is mutated in most head and neck tumors, the retinoblastoma (*Rb*) gene, a critical target of HPV-mediated transformation of virtually all cervical cancers, remains functionally active in many, if not most, oral cancers [29,30]. Furthermore, inducing an increase in *p21 *expression and activity, thereby influencing *Rb *phosphorylation and cell cycle arrest, has been demonstrated to significantly alter cell proliferation of SCC-15 [31], SCC-25 [32,33], and CAL27 cells [32] independent from *p53 *expression or activation. These studies suggest that modulation of *Rb*-specific pathways by the HPV E6 and E7 genes in these cells could be partially responsible for lowering the barriers to G1/S cell cycle progression of these cells, thereby increasing proliferation and cell viability in some cases.

Our experimental results examining the changes to cell viability, and perhaps the influence of HPV to alter the susceptibility of these cell lines to apoptosis, suggests that deactivation of *Rb*-mediated pathways, which may still be active in these cells, may be responsible, in part, for the observed increases in cell viability, particularly among the CAL27 cell line. However, the differential response to HPV strains between SCC-15 and SCC-25 cells, not only in proliferation response but also in other cellular phenotypes such as viability and adhesion, may be more complicated than direct inhibition of *Rb*-specific pathways. Thus these differential responses may involve protein binding specificities between these two cell lines for HPV16 or HPV18 gene products, or perhaps some other intrinsic difference or epigenetic parameter that has not yet been identified.

In fact, intrinsic differences among these cell lines have already been noted in some previous studies [32,34]. For example, although some of the major target genes altered during oral carcinogenesis may be common to these cell lines, the level of gene inactivation and mechanisms for this gene inactivation among specific tumor suppressors differs. For example, one study identified that p16/INK4A, an inhibitor of cyclin CDK and G1/S cell cycle progression, has been frequently reported as a target for inactivation in many oral cancers; however, SCC-25 cells were found to contain a homozygous deletion of p16/INK4A, while SCC-15 cells demonstrated hypermethylated promoter deactivation of this gene, suggesting that even the mechanisms involved in oral carcinogenesis among these cell lines are not equivalent [34]. Further research may lead to the identification of specific markers that will further our understanding of tumor-specific prognosis and treatments.

## Conclusion

Although evidence suggests that high-risk HPV may induce some percentage of oral carcinogenesis, more evidence is now emerging that HPV infection may also have the potential to significantly alter oral cancer proliferative phenotypes and outcomes. Determining the potential of HPV to alter phenotypic behaviors of already transformed oral carcinomas has thus become an important step in determining more accurately the prognosis and treatment options for patients with oral cancer. Thus, these data, which reveal differential responses to specific HPV strains among oral cancers, may be the first step to identifying the important molecular markers and pathways that could be used to determine more effective and appropriate treatment plans for oral cancer patients with concomitant oral HPV infections. We suggest that further research in these areas should yield additional information to help oncologists and researchers establish a rubric for generalizing the effects and most effective treatment options for oral cancer patients.

## Methods

### Cell culture

The human OSCC cell lines used in this study, CAL27, SCC-15, and SCC-25, were obtained from American Type Culture Collection (ATCC: Manassas, VA). CAL27 cells were maintained in Dulbecco's Modified Eagle's Medium (DMEM) with 4 mM L-glutamine, adjusted to contain 3.7 g/L sodium bicarbonate and 4.5 g/L glucose from Hyclone (Logan, UT). SCC-15 and SCC-25 cells were maintained in a 1:1 mixture of DMEM and Ham's F12 medium with 2.5 mM L-glutamine, modified to contain 15 mM HEPES, 0.5 mM sodium pyruvate, and 1.2 g/L sodium bicarbonate (ATCC), supplemented with 400 ng/ml hydrocortisone from Sigma-Aldrich (St. Louis, MO). Media for all cell lines was supplemented with 10% fetal bovine serum (FBS), and with 1% Penicillin (10,000 units/mL)-Streptomycin (10,000 μg/mL) solution (HyClone). Cell cultures were maintained in 75 cm^2 ^BD Falcon tissue-culture treated flasks (Bedford, MA) at 37°C and 5% CO_2 _in humidified chambers.

### Proliferation

Proliferation assays were performed of CAL27, SCC-15, and SCC-25, transfected with HPV16, HPV18, and co-transfected with HPV16/18, mock-transfected controls, and un-transfected controls. Proliferation assays were performed in the appropriate complete media in Corning Costar 96-well assay plates (Corning, NY) at a concentration of 1.2 × 10^4 ^cells per well, and proliferation was measured over three days. Cultured cells were fixed after 24 hrs (day 1), after 48 hrs (day 2), and after 72 hrs (day 3) using 50 μL of 10% buffered formalin, and were stained with crystal violet 1% aqueous solution (Fisher Scientific: Fair Lawn, NJ). The relative absorbance was measured at 630 nm using a Bio-Tek ELx808 microplate reader (Winooski, VT). Data were analyzed and graphed using Microsoft Excel (Redmond, WA) and SPSS (Chicago, IL). Three separate, independent replications of each experiment were performed.

### Viability

Prior to plating cells for adhesion and proliferation assays, aliquots of trypsinized cells were stained using Trypan Blue (Sigma: St. Louis, MO), and live cells were enumerated by counting the number of Trypan-blue negative cells using a VWR Scientific Counting Chamber (Plainfield, NJ) and a Zeiss Axiovert 40 inverted microscope (Gottingen, Germany). At each time point (day 1–3), several wells were processed using the Trypan stain, and live cells were enumerated using this procedure [35,36].

### Adhesion

Cell adhesion assays were performed of CAL27, SCC-15, and SCC-25, transfected with HPV16, HPV18, and co-transfected with HPV16/18, mock-transfected controls, and un-transfected controls. Adhesion assays were performed as previously described [37,38] in uncoated Corning Costar 96-well assay plates (Corning, NY) at a concentration of 1.2 × 10^5 ^cells per well (100 μL of 1.2 × 10^6 ^cells/mL solution) suspended in serum-free DMEM with no additives. Cells were allowed to attach for 30 min at 37°C with one modification. The modified adhesion assays used in this study eliminated the plate suspension step, in which non-adherent cells are generally removed by suspending the plate upside-down in a rotating tank of PBS. Following the incubation period, the cells were fixed using 50 μL of 10% buffered formalin and were subsequently stained with crystal violet 1% aqueous solution (Fisher Scientific: Fair Lawn, NJ). The relative absorbance was then measured at 630 nm using a Bio-Tek ELx808 microplate reader (Winooski, VT). Data were analyzed and graphed using Microsoft Excel (Redmond, WA). Three separate, independent replications of these assays were performed.

### Morphology

The number and percent of spreading and non-spreading cells were determined for each of the experimental and control cell lines in the adhesion assays, and for each day and condition in the proliferation assays. To accomplish this, cells were fixed in 50 μL of 10% buffered formalin, and were stained with crystal violet 1% aqueous solution (Fisher Scientific: Fair Lawn, NJ). The number and percent of spreading and non-spreading cells were then determined by visual inspection using a Zeiss Axiovert 40 inverted microscope (Gottingen, Germany) and confirmed with digital capture and Adobe Photoshop (San Jose, CA) Image Analysis tools.

### Transfection

CAL27, SCC-15, and SCC-25 cells were seeded in 25 cm^2 ^BD Falcon tissue-culture treated flasks in appropriate media as described above and allowed to achieve 70% confluence. Cells were then transiently transfected by adding 1 μg/mL of the full-length HPV type 16, cloned into the pBluescript SK-vector (ATCC #45113) and/or the HPV type 18, cloned into the pBR322 vector (ATCC #45152). These cell lines were also co-transfected with 1 μg/mL of HPV16 and 1 μg/mL of HPV18. The transfections were performed using the Stratagene Mammalian Transfection Kit (La Jolla, CA) according to the manufacturer's recommended protocol for CaPO_4 _transfection. Mock transfectants (mTF) of these three cell lines were also established by performing the same transfection protocol, but without using virus.

### RT-PCR

RNA was isolated from 1.5 × 10^7 ^cells of each of the experimental and control cell lines, using ABgene Total RNA Isolation Reagent (Epsom, Surrey, UK) and the procedure recommended by the manufacturer. RT-PCR was performed with the ABgene Reverse-iT One-Step RT-PCR Kit (ReadyMix Version) and a Mastercycler gradient thermocycler (Eppendorf: Hamburg, Germany) using the following primers synthesized by SeqWright (Houston, TX): HPV18 forward primer, ATGGCGCGCTTTGAGGATCC; HPV18 reverse primer, GCATGCGGTATACTGTCTCT; HPV16 forward primer, ATGTTTCAGGACCCACAGGA; HPV16 reverse primer, CCTCACGTCGCAGTAACTGT. One μg of template RNA was used for each reaction. The reverse transcription step ran for 30 min at 47°C, followed by denaturation for 2 min at 94°C. Thirty-five amplification cycles were run, consisting of 20 sec denaturation at 94°C, 30 sec of annealing at 58°C, and 6.5 min of extension at 72°C. Final extension was run for 5 min at 72°C. Reaction products were separated by gel electrophoresis using Reliant 4% agarose gels (Cambrex: Rockland, ME). Bands were visualized by UV illumination of EtBr-stained gels and captured using a Kodak Gel Logic 100 Imaging System and 1D Image Analysis Software (Eastman Kodak: Rochester, NY).

### Statistics

The differences between treatments were measured using a *t *distribution, α = .05. All samples were analyzed using two-tailed *t *tests as departure from normality can make more of a difference in a one-tailed than in a two-tailed *t *test. As long as the sample size is even moderate (>20) for each group, quite severe departures from normality make little practical difference in the conclusions reached from these analyses [39]. To confirm the effects observed from these experiments, further analysis of the data was facilitated with ANOVA using SPSS (Chicago, IL). Significance for ANOVA was 0.05.

## Competing interests

The author(s) declare that they have no competing interests.

## Authors' contributions

KK conceived, monitored, and coordinated the experimental design. NR, TC, AB, DJ, DJ, and SO carried out the microscopy and *in vitro *assays. Both KK and SO contributed equally to the writing of this manuscript.
